# In vitro testing of cyanoacrylate tissue adhesives and sutures for extracorporeal membrane oxygenation cannula securement

**DOI:** 10.1186/s40635-020-00365-5

**Published:** 2021-01-04

**Authors:** India Pearse, Amanda Corley, Nicole Bartnikowski, John F. Fraser

**Affiliations:** 1 Critical Care Research Group, The Prince Charles Hospital and University of Queensland, Brisbane, Australia; 2 School of Chemistry, Physics and Mechanical Engineering, Science and Engineering Faculty, Queensland University of Technology, Brisbane, Australia; 3 Adult Intensive Care Services, The Prince Charles Hospital, Brisbane, Australia

**Keywords:** Extracorporeal membrane oxygenation, Securement, Cyanoacrylate, Tissue adhesive, Sutures

## Abstract

**Background:**

Extracorporeal membrane oxygenation (ECMO), an invasive mechanical therapy, provides cardio-respiratory support to critically ill patients when maximal conventional support has failed. ECMO is delivered via large-bore cannulae which must be effectively secured to avoid complications including cannula migration, dislodgement and accidental decannulation. Growing evidence suggests tissue adhesive (TA) may be a practical and safe method to secure vascular access devices, but little evidence exists pertaining to securement of ECMO cannulae. The aim of this study was to determine the safety and efficacy of two TA formulations (2-octyl cyanoacrylate and *n*-butyl-2-octyl cyanoacrylate) for use in peripherally inserted ECMO cannula securement, and compare TA securement to ‘standard’ securement methods.

**Methods:**

This in vitro project assessed: (1) the tensile strength and flexibility of TA formulations compared to ‘standard’ ECMO cannula securement using a porcine skin model, and (2) the chemical resistance of the polyurethane ECMO cannulae to TA. An Instron 5567 Universal Testing System was used for strength testing in both experiments.

**Results:**

Securement with sutures and *n*-butyl-2-octyl cyanoacrylate both significantly increased the force required to dislodge the cannula compared to a transparent polyurethane dressing (*p* = 0.006 and *p* = 0.003, respectively) and 2-octyl cyanoacrylate (*p* = 0.023 and *p* = 0.013, respectively). Suture securement provided increased flexibility compared to TA securement (*p* < 0.0001), and there was no statistically significant difference in flexibility between 2-octyl cyanoacrylate and *n*-butyl-2-octyl cyanoacrylate (*p* = 0.774). The resistance strength of cannula polyurethane was not weakened after exposure to either TA formulation after 60 min compared to control.

**Conclusions:**

Tissue adhesive appears to be a promising adjunct method of ECMO cannula insertion site securement. Tissue adhesive securement with *n*-butyl-2-octyl cyanoacrylate may provide comparable securement strength to a single polypropylene drain stitch, and, when used as an adjunct securement method, may minimise the risks associated with suture securement. However, further clinical research is still needed in this area.

## Background

Extracorporeal membrane oxygenation (ECMO) is an invasive mechanical therapy used to provide cardio-respiratory support to critically ill patients when the native organs have failed [1]. ECMO therapy is delivered via large-bore cannulae [1], and the success of the therapy is, in part, reliant on adequate dressing securement of these cannulae. Effective securement, both at the insertion site and along the length of the cannula, may reduce the significant clinical risk posed by cannula migration, dislodgement or complete decannulation, which can lead to potentially devastating patient outcomes.

Cannula migration or movement can result in decreased circuit flow, compromising the effectiveness of ECMO support delivered [2, 3]; and increased turbulent blood flow resulting in haemolysis and potentially renal dysfunction [2, 4]. Cannula dislodgement may lead to catastrophic patient consequences due to loss of ECMO support, air entrainment and massive blood loss [5], and can be life-threatening [3]. To prevent these complications, clinical practice guidelines from the Extracorporeal Life Support Organisation state that ECMO cannulae must be effectively secured to the skin in at least two locations, with fixation and positioning of the cannulae checked at frequent intervals [6].

The true incidence of cannula malposition has not been quantified, but in a recent global survey of ECMO practices 34% of respondents stated that an adverse patient event had occurred in the last five years as a result of cannula malposition or dislodgement [7]. One-third of these cannula malpositions were directly attributed to suboptimal cannula securement [7]. This indicates that there is an urgent clinical need to improve methods used to secure ECMO cannulae at the insertion site, which is particularly relevant given the current management practices of less sedation [8] and increased mobilisation [9] in patients receiving ECMO. Due to the paucity of research in this area, there are currently no standardised, evidence-based clinical practice guidelines informing ECMO cannula dressing and securement practice. As a result, there is wide variation in cannula securement practices globally [7] and no ECMO-specific evidence regarding the most effective dressing and securement method.

Medical-grade cyanoacrylate TAs were first developed in 1949 and have been used for closure of both superficial lacerations [10, 11] and surgical wounds [12], and there is  also evidence for use in small-bore intravascular catheter securement [13–20]. However, there is currently very little evidence pertaining to the use of TAs to secure ECMO cannulae in the literature. TA may provide a potential adjunct method to secure ECMO cannulae [2] by reducing the need for invasive suturing, thereby lessening associated complications such as refractory suture site bleeding [2] and bloodstream infection through the creation of additional portals of entry for bacteria [21]. In a recent in vitro study, a ‘first-generation’ TA consisting of *n*-butyl-2-cyanoacrylate (Histoacryl®, B. Braun, Melsungen, Germany), demonstrated increased pull-out force required to dislodge the cannula at the insertion point when compared with a polyurethane dressing [2]*.* However, while older TA formulations, such as Histoacryl and other *n*-butyl-2-cyanoacrylate TAs, have a high bonding strength, they are prone to becoming brittle [10, 22–24], especially with repeated ‘top-up’ applications which can lead to adverse skin events such as skin tears [14, 16]. More recent formulations, comprising 2-octyl cyanoacrylate and *n*-butyl-2-octyl cyanoacrylate, claim to be more flexible whilst maintaining high tensile strength [22], but there is little independent evidence testing these claims. Therefore, it is important to test the ‘new generation’ TAs to determine if they offer a more clinically useful alternative to the older *n*-butyl-2-cyanoacrylate TAs for securing peripheral ECMO cannulae at the insertion site.

Given the clinical imperative that ECMO cannula remain well-secured and well-positioned, we aimed to determine the safety and utility of two TAs for peripheral ECMO cannula securement; and to test these against two standard methods of cannula securement—sutures and a transparent polyurethane dressing alone. Additionally, we aimed to determine the chemical resistance of the polyurethane ECMO cannulae to the TA formulations.

## Methods

This in vitro project was divided into two parts assessing: (1) tensile strength and flexibility of two TA formulations, sutures and transparent polyurethane dressing alone for peripherally inserted ECMO cannulae at the insertion site, and (2) assessment of the chemical resistance of the polyurethane ECMO cannulae to TAs.

### Strength and flexibility testing

Sections of porcine skin, 13 cm × 13 cm, were cut from the underbelly of recently euthanized adult pigs used in another research project. Hair was removed from the skin using surgical clippers and skin sections were mounted onto a purpose-built plastic securement frame. Once securely fixed, a 15-cm section of 23F Bio-Medicus femoral venous cannula (Medtronic Inc., Minneapolis, MN, USA) was inserted through the skin using a modified Seldinger technique, and the cannula secured to the surrounding skin at the insertion point with one of the following four securement combinations: (1) polyurethane dressing (PU dressing) (Opsite™, Smith and Nephew, London, UKTD); (2) sutures and PU dressing; (3) SurgiSeal® TA (Adhezion Biomedical, Wyomissing, PA, USA) (2-octyl cyanoacrylate) and PU dressing, or 4) Glubran ® Tiss2 TA (GEM, Italy) (*n*-butyl-2-octyl cyanoacrylate) and PU dressing. Two to three drops of TA were applied to the cannula insertion site, and dressings were left for five min after application before testing was commenced. Cannula sections were sutured with a 3.0 polypropylene suture using a drain stitch technique.

**Fig. 1:**
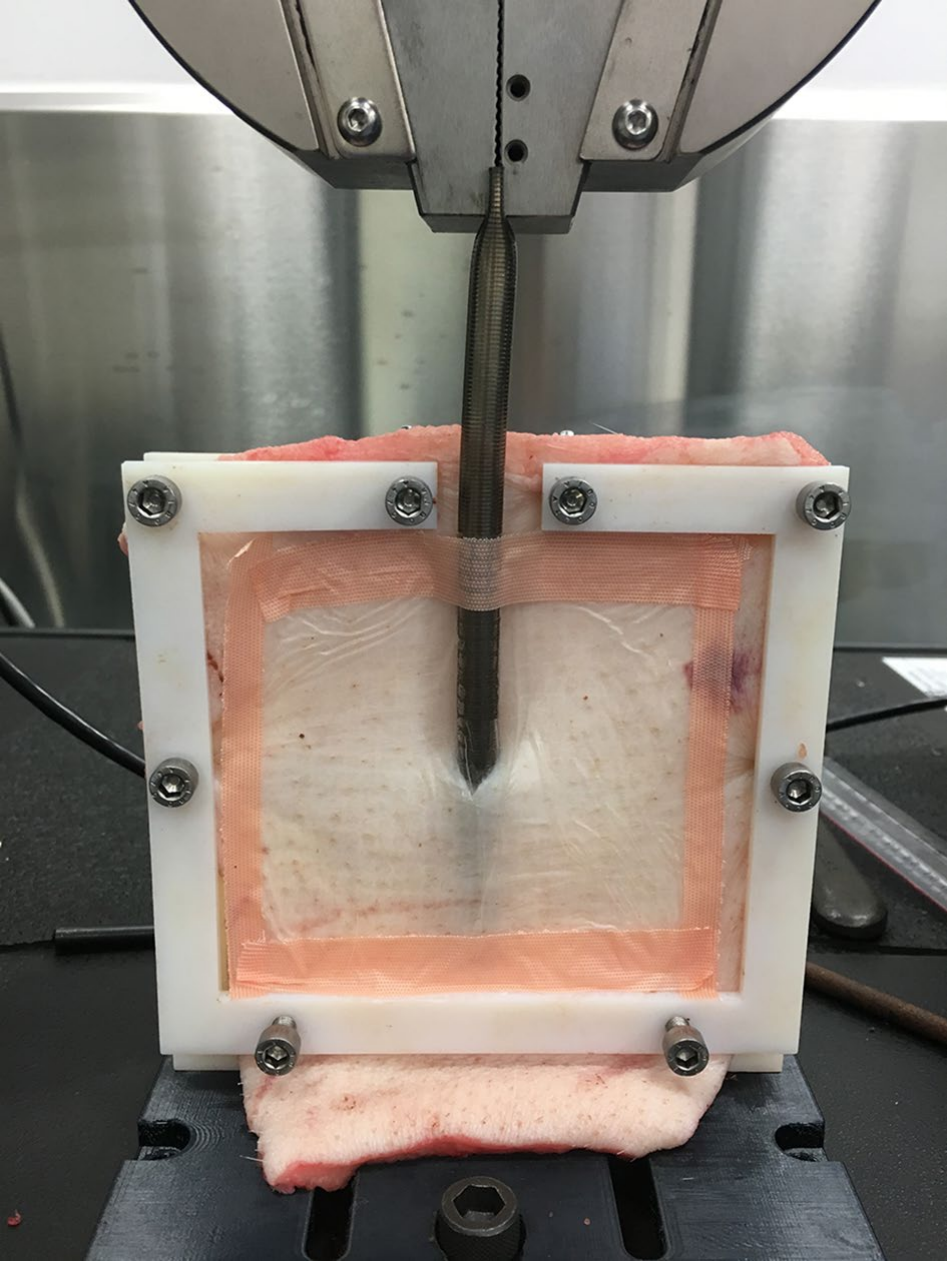
Secured cannula on the Instron 5567 Universal Testing System

The prepared frames were attached to the Instron 5567 Universal Testing System (see Fig. 1), which was then calibrated and ‘zeroed’, before the cannula was pulled from the skin at a rate of 250 mm/min. The maximal load (*N*) required for the securement bond to fail, and total distance of skin ‘tenting’ or ‘flexibility’ (mm) at the time of bond failure, was recorded for each securement method. Six individual tests for each securement method were completed.

### Chemical resistance testing

On 15-cm sections of 23F Bio-Medicus femoral venous cannula, TA was applied in a 1-cm band around the external surface of the mid-section. The TA was left to polymerise onto the cannula sections for 60 min. ‘Naked’ cannulae were also tested as a control. Each cannula section was mounted into the Instron 5567 before being subjected to pulling force at 250 mm/min. The maximal load (N) required to break the cannula in half, and total cannula extension (mm) at that time, was recorded for both control and TA cannula sections. Four control cannula sections, and five cannula sections each of 2-octyl cyanoacrylate TA and *n*-butyl-2-octyl cyanoacrylate TA were tested.

### Statistical analysis

All statistical analysis was performed using Prism 7.02 (GraphPad Software Inc.). Sample size calculations were based on similar work previously reported [2, 20]. Data were analysed using one-way ANOVA, followed by post hoc Tukey tests. Normally distributed data were reported as means and standard deviations (SD), while not normally distributed data were reported as medians and 25th and 75th percentiles. A *p* value of < 0.05 was considered statistically significant.

## Results

### Securement testing

Securement with sutures (34.35 N, SD 8.02) and *n*-butyl-2-octyl cyanoacrylate TA (35.51 N, SD 5.96) both significantly increased the force required to dislodge the cannula compared to a PU dressing alone (16.66 N, SD 5.56) (*p* = 0.006, *p* = 0.003, respectively). Both sutures and *n*-butyl-2-octyl cyanoacrylate TA also required a greater pull-out force than 2-octyl cyanoacrylate TA (19.68 N, SD 10.19) (*p* = 0.023 and *p* = 0.013, respectively) to dislodge the cannula from the skin. The pull-out force required to dislodge the cannula from the skin with PU dressing alone and 2-octyl cyanoacrylate TA did not significantly differ (*p* = 0.898).

Sutures (39.42 mm, SD 7.01) provided superior flexibility compared to a PU dressing alone (20.75 mm, SD 3.17), 2-octyl cyanoacrylate TA (10.26 mm, SD 6.49) and *n*-butyl-2-octyl cyanoacrylate TA (17.26 mm, SD 6.96) (*p* = 0.0004; *p* < 0.0001; *p* < 0.0001, respectively). There was no statistically significant difference in flexibility between 2-octyl cyanoacrylate TA and *n*-butyl-2-octyl cyanoacrylate TA (*p* = 0.254).

### Chemical resistance testing

There was no statistically significant difference in either tensile strength or flexibility of the cannula polyurethane after exposure to either 2-octyl cyanoacrylate TA (94.75 N (SD 5.45, *p* = 0.987) and 201.86 mm (SD 0.43, *p* = 0.468), respectively) or *n*-butyl-2-octyl cyanoacrylate TA (95.10 N (SD 6.58, *p* = 0.998) and 201.36 mm (SD 1.26, *p* = 0.654), respectively) for 60 min when compared to control (95.29 N (SD 0.91) and 200.05 mm (SD 3.89).

## Discussion

This in vitro study is the first to (1) directly compare tensile strength and flexibility of 2-octyl and *n*-butyl-2-octyl cyanoacrylate TAs for securement of ECMO cannulae, and (2) assess chemical resistance of cannula polyurethane against such TAs. *n*-Butyl-2-octyl cyanoacrylate TA demonstrated the highest force required to dislodge the cannulae, significantly higher than 2-octyl cyanoacrylate TA and PU dressing securement, and comparable to suture securement. *n*-Butyl-2-octyl cyanoacrylate TA also demonstrated a non-significantly higher degree of flexibility than 2-octyl cyanoacrylate TA, however both TAs were significantly less flexible than sutures and PU dressing securement. Cannula strength and flexibility were not affected by either TA. These findings suggest that TA, particularly *n*-butyl-2-octyl cyanoacrylate, may be potentially useful in the management of ECMO cannulae in terms of securement to prevent malposition or dislodgement.

The clinical applicability of our findings is multi-faceted. Firstly, ‘new generation’ TAs are flexible and strong enough to adequately hold catheters in place whilst accommodating natural movement of human tissue. Secondly, TA securement does not have the negative side effects associated with suture securement, such as bleeding, incidental perforation of intravascular device tubing [2], increased risk of bloodstream infection [21] and needlestick injuries [25]. Finally, emerging evidence suggests that cyanoacrylate TAs have favourable antimicrobial properties [2, 20, 26, 27]. These advantageous properties of TA may be translatable into ECMO cannula securement, but there is currently limited evidence with only one study currently published on the subject [2].

*n*-Butyl-2-octyl cyanoacrylate TAs have longer alkyl chains than 2-octyl cyanoacrylate TAs, resulting in more flexible bonds and a higher breaking strength [28, 29]. Our findings reflect this as *n*-butyl-2-octyl cyanoacrylate TA required significantly more force to dislodge the cannula compared to 2-octyl cyanoacrylate TA. The increased flexibility of *n*-butyl-2-octyl bonds also reduces the brittleness associated with older generation TAs [22, 23], and is also reflected in our findings. Given that *n*-butyl-2-octyl cyanoacrylate TA is stronger with similar flexibility to 2-octyl cyanoacrylate TA, *n*-butyl-2-octyl cyanoacrylate TA may be more suited to ECMO cannula securement, particularly in accommodating tissue movement whilst keeping the cannula secure and correctly positioned.

The results of the chemical compatibility testing also align with previously published literature [2, 30], which demonstrates no breakdown of the polyurethane ECMO cannulae after 60 min of exposure to *n*-butyl-2 cyanoacrylate TA [2], or in polyurethane peripherally inserted central catheters after 12 weeks of exposure to *n*-butyl-2-octyl cyanoacrylate [30]. Previous literature has also explored chemical resistance of ECMO cannulae against commonly used adhesive remover wipes (Remove™, Smith & Nephew, Mississauga, ON), finding the removal agent significantly weakened the cannula polyurethane after 60 min of exposure [2]. This finding is not problematic in cannulae being removed at ECMO discontinuation, however, for cannulae being repositioned but remaining in situ, the clinical significance of this finding requires more investigation. If adhesive remover wipes are used to dissolve TA on cannulae to be repositioned, a protocol must be in place to clearly outline how to effectively eliminate all adhesive remover residue on the cannulae. We consider our findings the first step in the body of evidence surrounding TA use in ECMO cannulae and acknowledge that it is imperative that further safety data be generated before TA may be considered for clinical use.

There is currently no published literature which has tested sutures against TA for the purpose of ECMO cannula securement, but sutures are routinely used in the securement of smaller-gauge intravascular devices [31]. A recent global survey of ECMO cannula management reported 93% of centres use sutures to secure ECMO cannulae [7] however, sutures are not standard clinical practice in our hospital. Furthermore, unpublished data from a recently conducted study of ECMO cannula management in Australia and New Zealand has highlighted only 50% of Australian sites routinely suture cannulae, and 73% of cannulae insertion sites are covered with a transparent dressing alone. Given this, we thought it imperative to assess the securement properties of sutures and transparent PU dressing as control, as these are widely used in Australia.

Flexibility of securements is important to accommodate the natural movement of human skin. This study demonstrated that suture securement provided the greatest degree of flexibility compared to all other securement options. However, the flexibility demonstrated by suture securement may not be true ‘flexibility’, but rather an initial uptake of slack in the suture on commencement of force testing. This resulted in a concerningly large (nearly 4 cm) amount of movement which, in the clinical setting, could result in significant cannula migration during transport of the patient on ECMO or patient repositioning whilst still appearing ‘secure’. This highlights that, despite frequency of use, sutures may not be the ideal ECMO cannula securement method, and further research is required in this area to test potentially safer options.

Patients receiving ECMO are at increased bleeding risk due to the anticoagulation required to maintain patency of the ECMO circuit [6, 32]. Cannula securement with sutures, which cause an additional source of bleeding, may contribute to overall blood loss during ECMO [33]. Tissue adhesive promotes insertion site haemostasis in smaller intravascular devices [16, 34, 35], and may have similar effects at ECMO cannulae insertion sites. However, TA may be unsuitable in patients actively bleeding from cannulation sites as inhibition of blood flow from the insertion site wound with TA may cause haematoma [36]. Furthermore, active bleeding from cannula insertion sites may inhibit effectiveness of the TA seal [19], a phenomenon described in central venous catheters (CVCs) where TA lost adherence with coagulopathic ooze, diaphoresis and hair re-growth at the insertion site [19]. For this reason, we suggest TA be used as an adjunct securement method, and not as the sole method of cannula securement.

The prevalence of ECMO cannula infection is estimated to be more than quadruple that of other intravascular devices (4.8 vs 1.2 episodes per 1000 ECMO days) [37] suggesting that cannula-related infection control strategies require further investigation. TA possesses broad-spectrum antimicrobial properties that could reduce entry of micro-organisms through the insertion site, thereby decreasing potential bloodstream infection [2]. Minimising cannula-related infections is of critical importance because cannulae, unlike other intravascular devices, cannot be routinely exchanged if infection is suspected as doing so poses significant risk to the patient [6, 38]. Furthermore, evidence suggests TAs have both bacteriostatic and bactericidal effects on skin micro-organisms commonly responsible for ECMO-related infections [2, 20, 22, 26], but is dependent on the integrity of the TA seal with the skin [39] which lasts 5–10 days [40]. As such, TA may be effective not only for securement, but also for infection inhibition in this patient population, provided TA is ‘topped up’ to maintain seal integrity.

There are limitations to this study. Both TA formulations were strength tested after one application to the cannula insertion site. The results may therefore not entirely reflect the strength of securement after the TA has been ‘topped up’. This study also only simulated ‘peripheral’ cannulation, as the use of TA to secure ‘centrally’ inserted ECMO cannulae (i.e. those through the open sternum of the patient) would not be appropriate. Additionally, while most ECMO cannulae used in clinical practice are made from polyurethane [41] some are made from other substances, and our results would not be translatable to these cannulae types. Finally, this study tested only one suturing technique, but it is acknowledged that suturing practice may vary significantly between centres and therefore impact on the strength and flexibility of securement.

## Conclusion

ECMO cannula securement with TA appears to be a promising adjunct to current insertion site securement practice, but requires further investigation. Tissue adhesive securement has similar securement abilities and strength to suturing and higher tensile strength than polyurethane dressing securement, however, TA properties and suitability vary between products and formulations. A randomised control trial in the clinical setting will definitively determine whether the pre-clinical results presented in this study can translate into an effective approach to reducing ECMO cannula migration, malposition or dislodgement and ECMO cannula-related infections.

## Data Availability

The datasets generated and analysed during this study are available from the corresponding author on reasonable request.

## Abbreviations

ECMO: Extracorporeal membrane oxygenation
TA: Tissue adhesive
